# Clay as Space for Negotiation: Gender Relations in Traditional Ceramic Production

**DOI:** 10.12688/f1000research.177142.1

**Published:** 2026-05-19

**Authors:** Nadia Sigi Prameswari, Erni Setyowati, Muhammad Nasirul Umam

**Affiliations:** 1 Visual Arts, Universitas Negeri Semarang, Semarang, Central Java, 50229, Indonesia; 2 Architecture, Universitas Diponegoro, Semarang, Central Java, Indonesia

**Keywords:** material culture; embodied knowledge; ceramics, gender relations

## Abstract

**Background:**

This study examines how traditional ceramic production practices in Kasongan are formed through interactions between the artisan body, clay materials, gender relations, and community social structures. This study analyzes how traditional ceramic production practices in Kasongan, Yogyakarta, are shaped by the interaction between the artisan body, clay materials, gender relations, and the social structure of the community.

**Method:**

A descriptive–interpretive qualitative approach was used with thematic-inductive analysis. Data obtained through semi-structured interviews, participatory observations, and visual documentation of six purposively selected artisans were analyzed.

**Results:**

The results of the study show that the body becomes the main source of knowledge as skills grow through physical experience and repetitive work rhythms. The involvement of the body with clay forms an affective relationship in which material is understood as a living partner that influences the creative process. Such a relationship forms an aesthetic born from social collaboration and the negotiation of production and market needs. The production space becomes a fluid arena for gender negotiation through daily practice without a rigid division of roles. The contribution of this research lies in the new understanding that the tradition of craft survives through embodied negotiations that continue to occur in everyday practice. The aesthetics and sustainability of Kasongan ceramics result from social negotiations involving bodies, materials, gender, traditions, and market demands.

**Conclusion:**

The production space serves as a flexible social arena with a division of labor not defined by gender, but by production habits and needs. The entire process shows that Kasongan is a living ecosystem of cultural knowledge that survives through embodied practices, solidarity, and adaptability.

## 1. Introduction

Ceramics is one of the craft arts where traditional, local knowledge, and cultural memory serve to support social structure in society. No exception is placed on Indonesian society in relation to the practice of ceramics as an economic production, as well as a medium for cultural representation that represents social relations, symbolic values, and communal identity. Kasongan, like other Yogyakarta’s ceramic producing centers, displays its legacy through family practices in the production and transmission of skill generation to generation, sharing the roles of men and women in the activities ( Suharson, 2024). This practice reflects that the ceramicist cannot be cut off from the dynamics of material culture and social relationships that nurture family members.

At a global level, studies on craftsmanship argue that the phenomenon of ‘craft’ cannot be analyzed simply in terms of production techniques. For example, Sennett (2008) views skills as a type of knowledge connected to the body, which is associated with reconstruction being closely linked to rhythm, visual workout, and bodily feeling. Further, Ingold (2013) highlights that there is a dialogue between the craftsman and the material, and therefore, creativity arises through continual interactions of the body with the material. In gender theory, theorists Butler (2011) and Connell (2020) state that social acts and habits depict the form of genre. The framework opens the possibility of understanding the practice of craft not only as a technical activity, but also as an arena of gender performativity. Although the theoretical framework is developing, research on traditional Indonesian ceramics still focuses on preserving traditions, production techniques, business efficiency, and design innovation ( Khumairoh et al., 2025). Aesthetic studies, such as those conducted by Katoppo (2023), tend to discuss the form and symbolism of the work, but have not studied much about how the body, work experience, and gender relations play a role in shaping these aesthetics. Such a trend found in previous research suggests that the dimensions of the body, emotions, and gender as integral parts of craft practice still go unnoticed by academia, although global literature has emphasized the importance of these dimensions in understanding material cultural practices.

The gap is increasingly wider with respect to the roles of men and women in ceramic production, which tends to be stereotypically understood in popular discourse and cultural policy, without a scientific analysis of how the division of labor is formed, negotiated, and practiced in everyday life. Craftsmen are seen as homogeneous entities and do not distinguish how body experiences, work strategies, and emotional relationships with materials differ between men and women. In addition, although the aesthetics of Kasongan ceramics are widely discussed in the context of form innovation or local identity, no studies have placed it as a product of social negotiation that takes place in the production space. This research gap indicates that there is a necessity for a study that could systematically relate the concepts of embodied knowledge, material culture, and gender performativity in comprehending traditional Indonesian ceramic practices. Such research is significant because it demonstrates how identity, knowledge, and aesthetics are often produced not only through techniques, but also through bodily experiences, social habits, and relations to materials. This approach can further provide insights into how craft communities respond to social and economic change and the role gender relations play in ensuring the sustainability of craft traditions. Thus, this research makes an innovative contribution by bringing into light an analysis that views ceramic production practices as dynamic gender negotiations. The novelty of the research is that it seeks to integrate theory of embodied knowledge, material culture, combined with gender performativity to “read” function (work-processes), body-strategies and construction in determining aesthetics as welll identity of Kasongan ceramics. This trajectory not only extends the range of what can be learned about Indonesian craft arts but also contributes a theoretical angle to gendering and material culture analysis in traditional societies in Indonesia.

This study used an interpretive descriptive qualitative approach with an intrinsic case study design. This approach was chosen because the focus of the research lies in an in-depth understanding of traditional Kasongan ceramic production practices as a complex cultural phenomenon involving the interaction between the artisan’s body, materials, and gender construction. Intrinsic case studies are used when the phenomenon being studied is distinctive and not intended for broad generalization, but rather for the exploration of meaning in a particular context ( Stake, 1995). This approach is in line with the research objectives, theoretically, this research is based on the theory of gender performivity ( Butler, 2011) and the concept of craftsmanship ( Sennett, 2008), which together explain that work practices are not neutral activities, but rather a means by which social and cultural identities are expressed. From this point of view, the differences between men and women in the production of ceramics are not only a technical aspect but also a social process that shapes the aesthetics and cultural character of a work. This study aims to explain how gender shapes work patterns, works, and symbolic values in traditional ceramic art. The findings of this study are expected to contribute to the study of craft arts and gender studies in Indonesia, while affirming that the differences in creative expression between men and women are part of the socio-cultural diversity that needs to be understood and acknowledged.

## 2. Materials and methods

The research will be carried out in November 2025 at the Kasongan Handicraft Tourism Village, Kasihan District, Bantul, in the Special Region of Yogyakarta. This area was chosen purposively because it is a ceramic center that maintains a family based production system, inheritance of techniques across generations, and the involvement of men and women in all stages of production. The long history of Kasongan as a center of pottery since the Islamic Mataram period supports the relevance of the location as a research context for traditional craft research in Romadoni and Pranoto (2023).

Table 1 shows the informants were selected using purposive sampling ( Patton, 2002) with the following criteria: 1) actively working as craftsmen for at least five years, 2) directly involved in the production process, and 3) willingness to participate in interviews and observations. Six informants were selected based on the principle of data saturation ( Guest, Bunce & Johnson, 2006). They represent a variety of genders, ages, and work experiences, thus providing a comprehensive overview of the ceramic production practices in Kasongan. The informant data are shown in the following table:

**Table 1.** Research informant profile.

No.	Informant code	Gender	Age	Duration of work
1.	P1	Female	49 years old	16 years
2.	P2	Male	26 years old	5 years
3.	P3	Male	70 years	20 years
4.	P4	Male	26 years old	8 years
5.	P5	Female	45 years old	28 years
6.	P6	Female	40 years	17 years

Data collection was performed using three main techniques, each of which complements each other to increase the credibility of the data. First, the interview was carried out in person with flexible open-ended question guildelines. It is a methodological tool available to those who seek to understand experiences, perceptions, tacit knowledge, and how artisans make sense of their work. The latter method was selected because it can provide insights into subjective and reflexive dimensions ( Kvale & Brinkmann, 2009). Second, two weeks of participatory observation was conducted to document bodily strategies, working rhythms, techniques for shaping clay, social activity, and tool use. Participatory observation is a way for researchers to capture nonverbal, kinesthetic dimensions of being that are not always captured within the context of interviews ( Spradley, 1980). Third, documentation through photographs, production records, antique archives, and exhibition documentation has been documented to map the continuity of design, engineering alteration, and aesthetic dynamics in the making of Kasongan ceramics. The findings are also reinforced and contextualized with visual documentation, aiding the triangulation of data ( Wahidin, 2025). Informed consent was obtained from all participants prior to data collection. All participants were adults (≥18 years old). Participants were provided with detailed information regarding the objectives of the study, the voluntary nature of participation, and assurances of data confidentiality. Oral consent was obtained to ensure that all participants could understand the research information equally, without being hindered by reading ability or comprehension of written documents.

The analysis was conducted using an inductive approach, referring to the Ridder model ( 2014). The data analysis process was conducted in three interrelated stages. First, data reduction was carried out by sorting out important data, grouping information based on relevance, and eliminating non-essential data to clarify the focus of the research. Second, thematic coding was used to identify patterns, categories, and themes, such as body strategies, division of labor, relationships with materials, and aesthetic orientation. This stage starts with open coding to capture the diversity of information and then continues with axial coding to build relationships between themes. Third, conclusions are drawn through a dialogical interpretation process with key theories such as gender performativity ( Butler, 2011), craftsmanship and embodied knowledge ( Sennett, 2008), and material engagement ( Ingold, 2013), all of which are analytical frameworks in the initial research document.

## 3. Results

**Figure 1.**
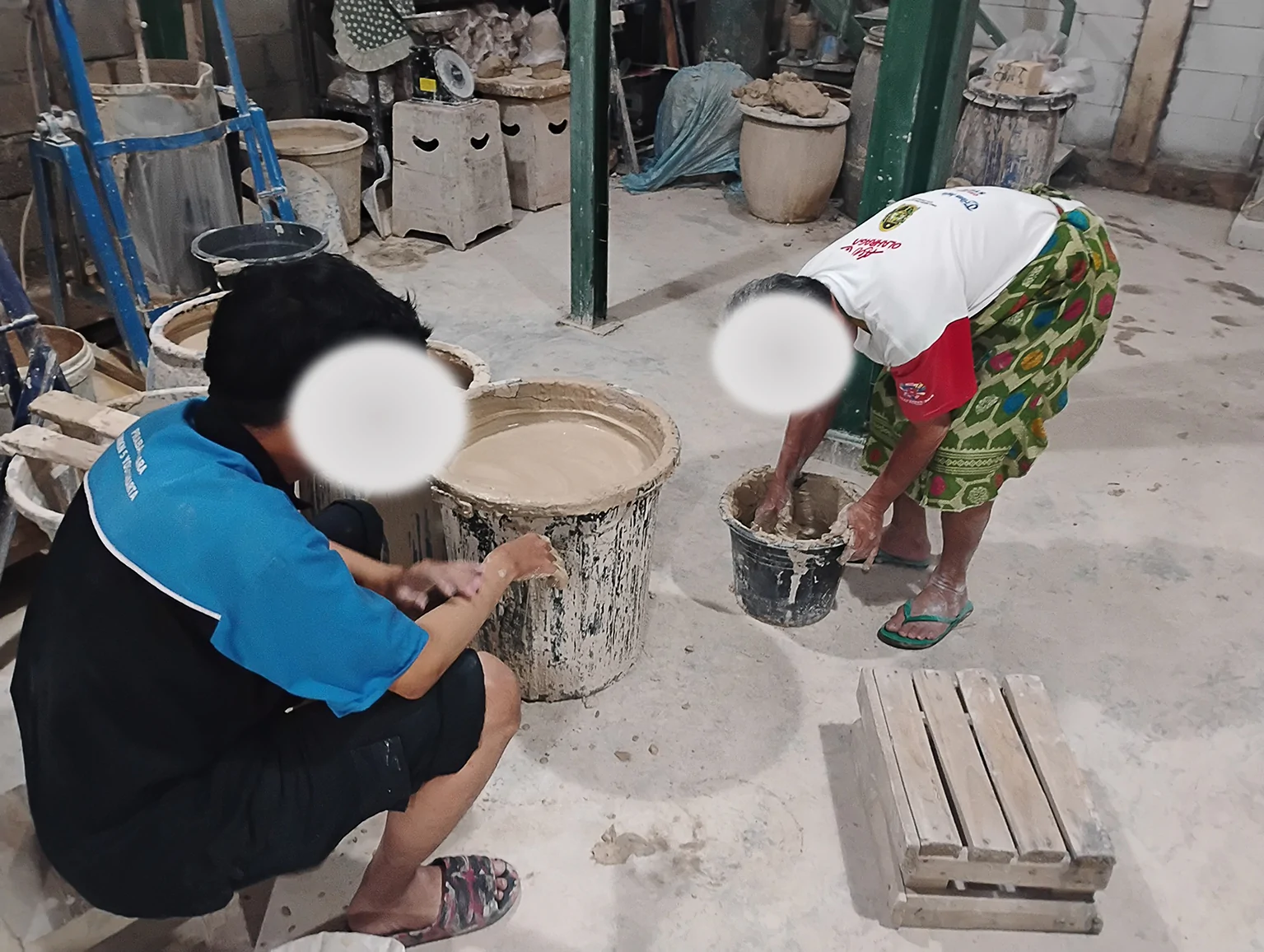
Material pouring process.

Analysis of observational data and field interviews showed that the body is the center of knowledge production in the practice of Kasongan ceramics. All informants, both male and female, explained the work process through references to body parts, rhythm of movement, position, physical endurance, and sensory responses. This pattern emphasizes that ceramic skills are not only technical abilities, but also embodied knowledge, which is knowledge that is formed through something abstract and concrete simultaneously into a person’s body, in this case between the body and material ( Rohman & Faizah, 2024). The emphasis on the body as an instrument as well as a reservoir of knowledge is in line with the concept of Sennett (2008), who states that craftsmanship does not only lie in the mind but also in the reflective work of the body that is formed in repetition and routine. This is also consistent with Ingold’s (2013) view that the creation of craft art objects is a process of correspondence, not domination: the craftsman’s body is constantly learning to read the material through the pressure, weight, resistance, texture, and moisture of the clay. Figure 1 illustrates artisans preparing raw materials for production.

The differences in body strategy between men and women emerged as a stable thematic pattern. Male craftsmen rely on high muscle strength, postural stability, and intense mobility, especially during the initial stages of formation, compaction, or removal of heavy materials. For example, P3 stated that:

*“Mind (concentration/solution), hands, and feet (mobility)”* (P3, November 17, 2025).

This shows that their physical work is not merely mechanical but involves coordination between mental focus, hand strength, and footwork rhythm as a form of body-cognition integration typical of craft. At the same time, men emphasized resilience to prolonged working positions. However, this must also be performed by women. P4, for example, stated that her body should last for seven hours in the same position.

“ *The body is not fit, production is slow, focus is reduced, and the effect of the final result of the work”* (P4, November 17, 2025).

This indicates that static resistance is a part of the workload during the formation or compaction stage.

In contrast, female craftsmen display different body strategies, emphasizing micromovements, rhythmic stability, and hand sensitivity. Women are dominant in tasks such as splicing, smoothing, finishing, and detail correction, which are stages that require sensory precision and gentle rhythm. This is in line with the expression of Indiworo (2016), which states that women have advantages such as diligence, meticulousness, tenacity, patience, honesty, toughness, a high sense of responsibility, strong will, high spirit, and discipline. However, P1 stated that the part of the body that often feels heavy is the waist because it is often used to lift the ground.

*“Waist, often used for lifting the ground*” (P1, November 17, 2025).

This suggests that although women’s work is often labeled as delicate, the physical load remains significant, especially in static and repetitive positions. These data illustrate that the body’s strategy is influenced not only by the division of labor but also by habitus: an internalized pattern of years of routine. This is in line with Bourdieu’s (1990) thought that the body stores social and technical memory through daily repetitions; Craftsmen no longer thought about how to move their hands because their bodies have memorized the work field.

In addition to the movement patterns, the forms of body fatigue also showed differences. Men tend to experience fatigue in shoulder, arm, and leg mobility, while women experience fatigue in the waist, forearms, and static positions. This information confirms the existence of a vertical, not hierarchical, division of physical burden and that fatigue is not just a side effect of work, but also part of the aesthetic production process. As P4 noted, suboptimal body conditions can slow production and affect the final result, which shows a close relationship between body condition and quality of work.

“ *The body is not fit, production is slow, focus is reduced, and the effect of the final result of the work”* (Erfina, November 17, 2025).

The results of the thematic analysis showed that both men and women viewed the body as the center of expertise, but their embodied experiences differed due to variations in the type of movement, rhythm, and intensity of work. This difference in the body’s strategy does not result in a rigid dichotomy but forms a natural specialization in the production process. At the operational level, the body’s strategy affects the stages of work, rhythm of production, and aesthetic characteristics of the Kasongan ceramics. This confirms that the body is not just a tool, but also an arena of meaning production: the body works, the body remembers, the body adapts, and the body shapes aesthetics. In harmony with the expression, the body will “remember” certain movements and be able to perform them automatically ( Pamungkas, 2025).

**Figure 2.**
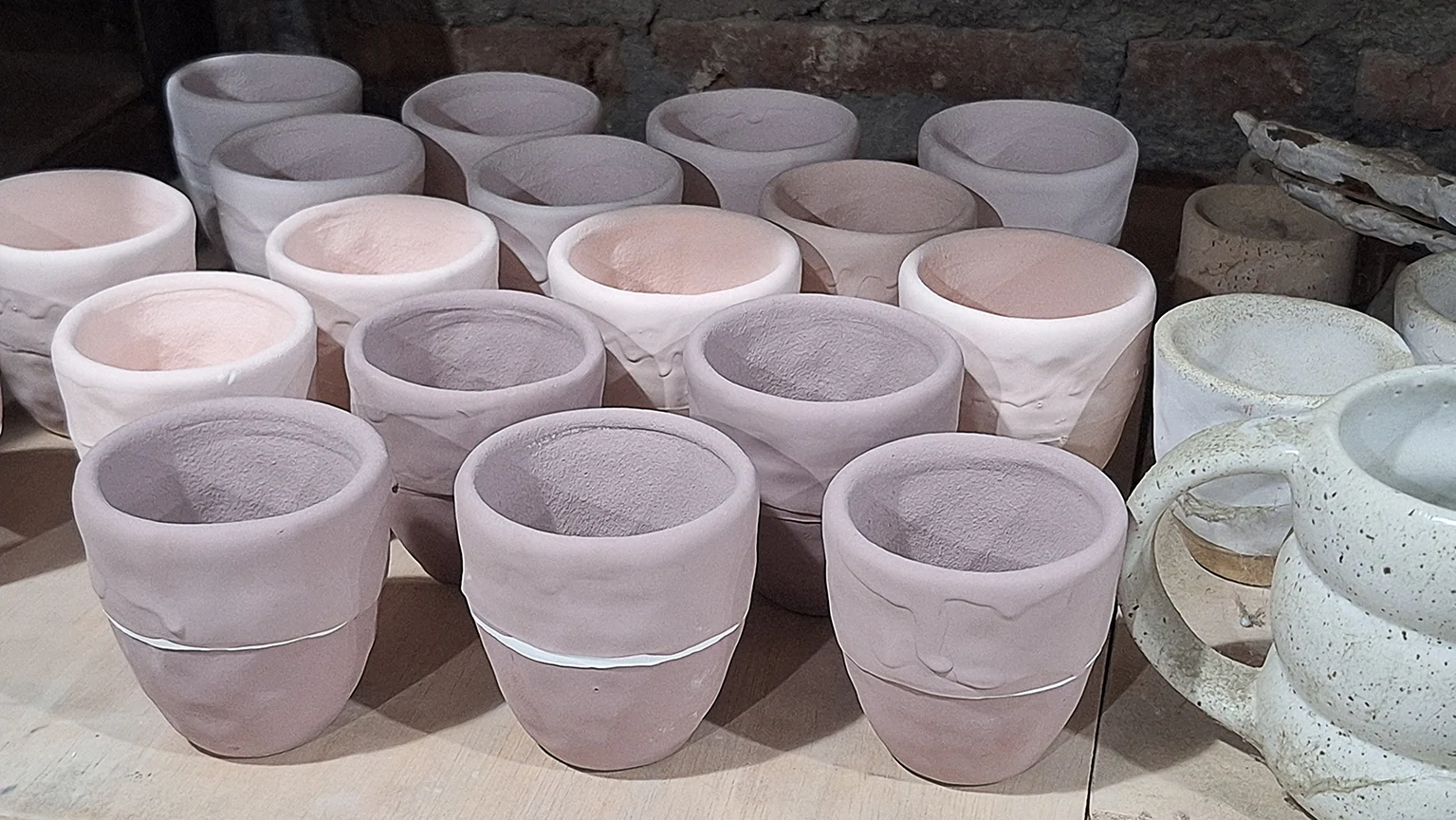
Ceramic vessels at the initial forming stage prior to drying and firing.

**Figure 3.**
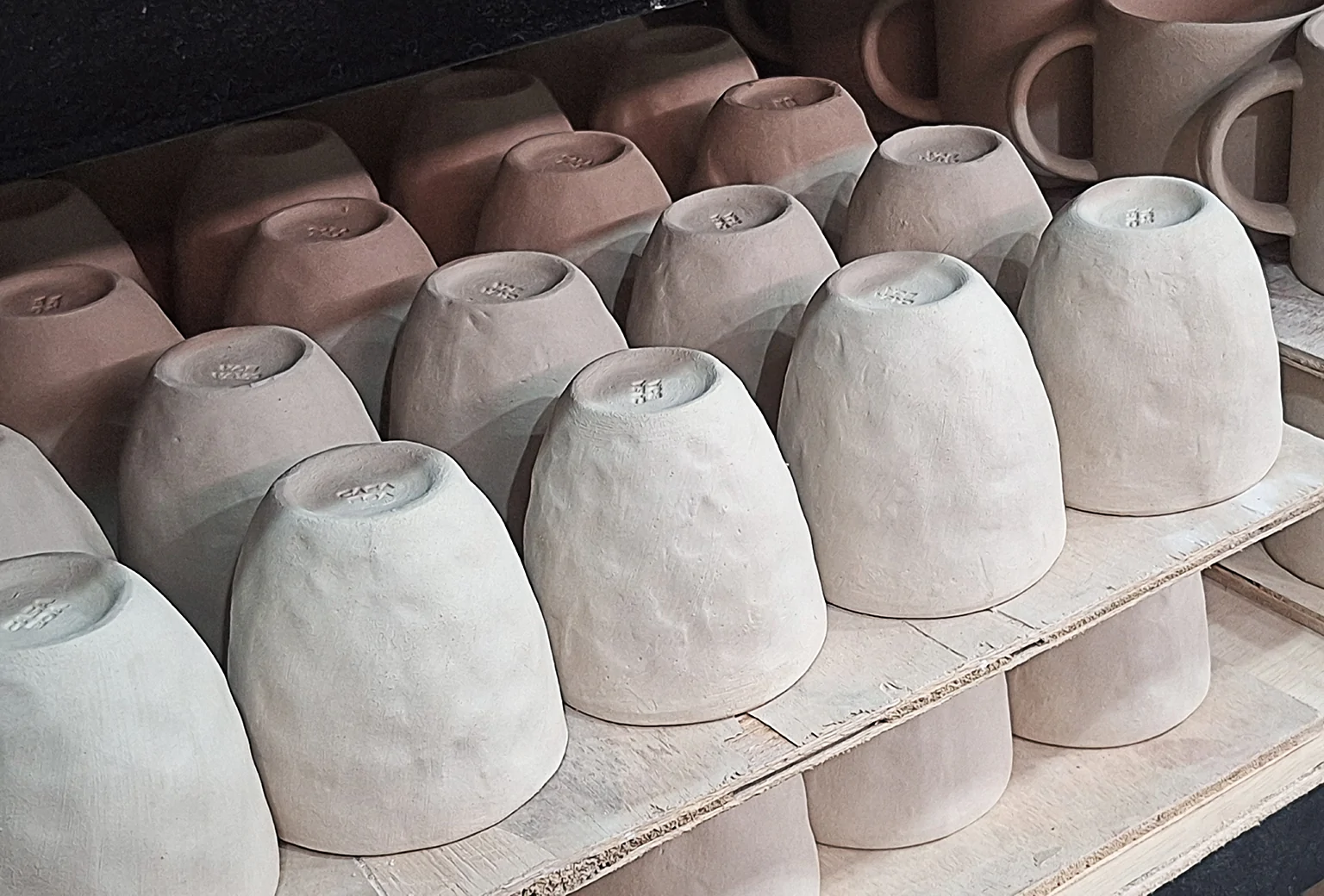
Ceramic vessels during the drying stage prior to firing (greenware condition).

Table 2 shows that thematic-inductive analysis of interview and observation data shows that clay in Kasongan ceramic practices is not understood as an inert object, but as an object that lives emotionally, symbolically, and morally for craftsmen. In the initial coding stage, various keywords emerged such as life, living, calm, healing, responsibility, and worship, which through the focused coding process narrowed down into a large category of affective relationships between craftsmen and materials. This is important because it shows that the practice of ceramics is not just a technical process of turning soil into a disposable or aesthetic object, but also an emotional experience that shapes the identity, daily life, and work orientation of the craftsman. Thus, clay is positioned not only as a material but also as a dialogical partner, in line with Ingold’s (2013) view that materials in traditional crafts have agency influencing the thoughts and actions of their makers. Most informants described the clay as alive. P6 stated that the land is alive and can be formed according to the will, which shows the perception that the land has energy, as well as a creative negotiation space between human will and material responses. This is evident in how the artisans exert maximum effort in creating the products, as illustrated in Figure 2 and Figure 3.

**Table 2.** Body strategy in ceramic production.

Subtheme	Description of findings	Field indicators
Macro Motion (Male)	Wide movement pattern, intense pressure, large muscle strength, high mobility	Early formation, soil compaction, material removal; hand-to-foot coordination; Strong hand pressure
Micromotion (Female)	Smooth movement, stable rhythm, high sensitivity	Refining, splicing, finishing; old static position; Consistent touch
Body Fatigue Center	Fatigue points differ by role	Male: shoulders, arms, legs → heavy work; Female: waist, forearm → repetitive work
Work Rhythm	Rhythm differences as a production strategy	Male: fast & efficient; Female: stable & repetitive
Body as Knowledge	The body becomes an archive of experience	The informant referred to the body directly as the center of work, not a secondary tool

“ *Land is alive, it can be shaped as we want”* (P6, November 17, 2025).

The same is affirmed by P4 through the statement that the land is alive and living, which shows the mutualistic relationship between craftsmen and materials as a source of life.

“ *The land lives and lives”* (P4, November 17, 2025).

At the stage of further analysis, a thematic pattern emerges that associates clay with emotional and therapeutic spaces. For some female craftsmen, working with soil is a way to cultivate feelings and relieve psychological stress. P1 stated that the activity of shaping soil can cause problems to disappear, indicating the role of clay as a medium of emotional healing, consistent with Leone (2020) research on pottery as a means of emotional reconstruction in traditional craft communities.

*“No, problems disappear if they are made to work”* (P1, November 17, 2025).

Touch of the material, rhythm of hand movements, and repetition of the process become meditative mechanisms that harmonize the body and mind. Meanwhile, male informants tended to associate clay with moral values and work ethics. Work ethics are formed from long-term cultural processes, and differences between communities occur due to different experiences, challenges, and cultural responses ( Febryani, 2022). In line with the P2’s expression, P2 emphasized the importance of maintaining professionalism even though he had personal problems, while P3 interpreted the activity of forming land as an activity worth worship. Despite their different orientations, these affective and moral dimensions are both sources of motivation and resilience in carrying out work.

**Figure 4.**
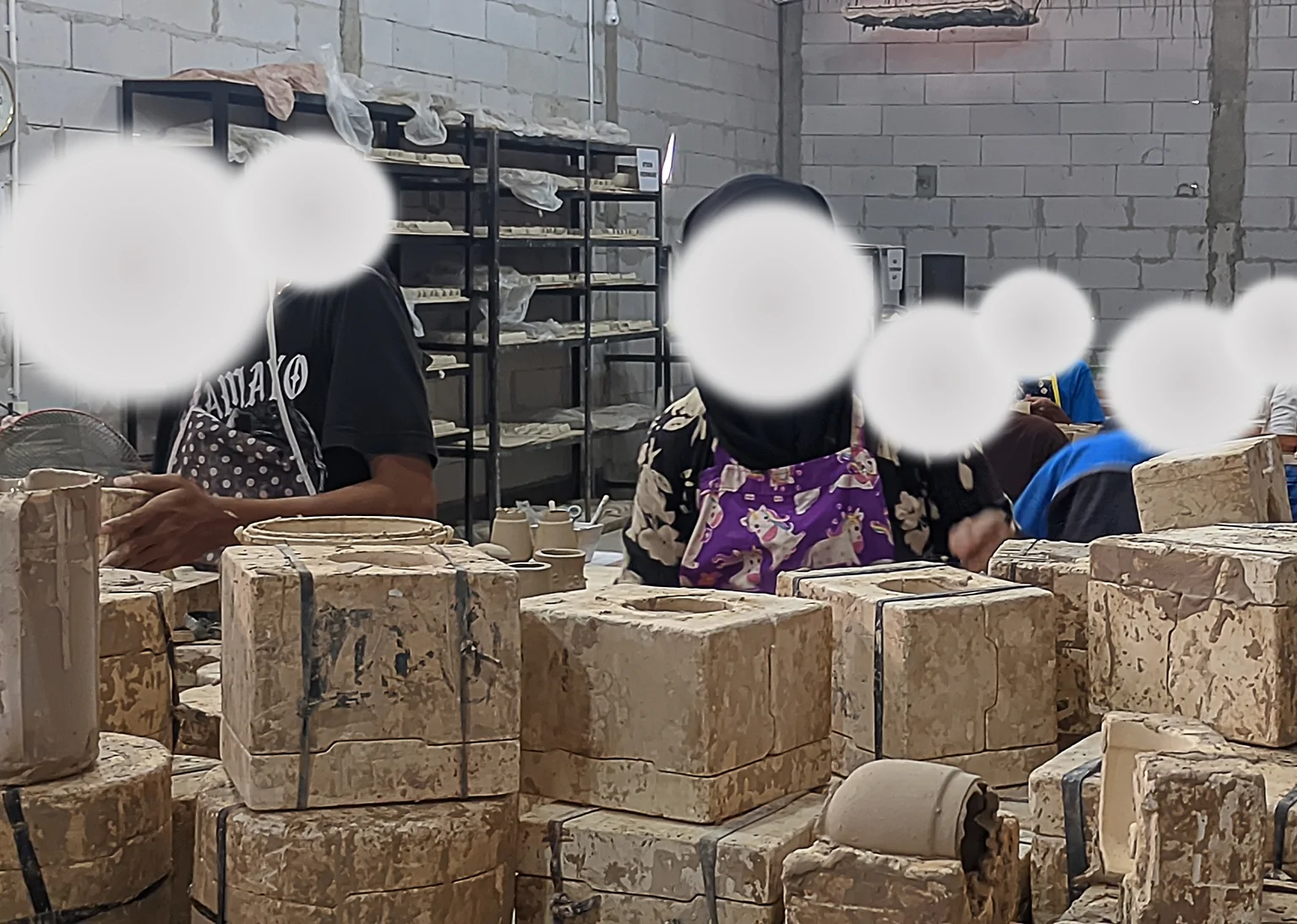
Fireplace process shape.

The relationship between senses and materials also appears to be significant in the formation of aesthetic value. Craftsmen distinguish between good and bad soils through hand experience, not through technical parameters, so that sensory experience plays a role in determining the shape, texture, thickness, and visual style of ceramics. Figure 4 shows that female craftsmen who work a lot in the finishing stage have an intense relationship with fine textures, whereas male craftsmen interact more with material resistance in the early formation stage. Emotional conditions are also seen as determinants of the quality of work ( Gani et al., 2018). As P4 revealed, when the body and mind are not fit, the process becomes slow and the result is not good. These findings confirm a causal relationship between body state, emotional state, and craft quality. Overall, the affective relationship between craftsmen and clay shapes the practice of Kasongan ceramics as a work that involves the simultaneity of physical, emotional, and spiritual dimensions, while showing that materials are not only objects of technical manipulation, but entities actively involved in the creation of form and meaning.

Table 3 shows that the results of the analysis of observations and interviews show that the aesthetics of Kasongan ceramics were not born as an individual expression of a craftsman but as a social product formed through body interactions, work habits, community provisions, and market demands. This arises from coding that groups keywords such as detail, precision, pressure, large, subtle, motif, rhythm, finishing, and *pakem*, which then fuses into one big theme about aesthetics because of inter-role negotiations rather than personal preference. These findings are in line with the view that the aesthetics of craft cannot be separated from embodied practice; that is, the way the body works and interacts with materials ( Rahmawati, 2018). When the male and female bodies have different movement strategies, these differences produce certain aesthetic tendencies, so that the aesthetics of Kasongan are not only a matter of design or form, but also the footprints of the craftsmen’s bodies that materialize as bodily imprints.

**Table 3.** Affective relationship to clay.

Subtheme	Description of findings	Field indicators
Land as a living entity	Material personified; understood as “living” and meaningful	Craftsmen use the terms *life*, *living*, *having a taste*
Land as an emotional space	Soil as a medium of healing and emotional escape	The informant said that work made the problem disappear; Calming work rhythm
Land as a moral ethos	Material is understood as a responsibility, worship, or call to work	Narrative of professionalism, spirituality, and dedication
Sensory relationships	Hands-on familiarity with texture, pressure, and soil temperature	“Good” or “not” evaluation based on touch
The effect of affection on quality	Emotions affect the precision and finish of the work	“Slower” or “less precise” production when emotional/unfit

Further analysis revealed that aesthetic differentiation based on body strategy emerged as a consistent pattern. Male craftsmen tend to produce large, firmly structured, symmetrical, and strong contours in harmony with macro motion patterns, the use of physical force, and intense hand pressure. By contrast, female craftsmen tend to produce works that are detailed, smoothly textured, have a soft visual rhythm, and are consistent in the finishing stage, which is related to micro-movement-based work strategies, smooth rhythms, and position stability. Although different, the two tendencies are not seen as hierarchical divisions but as a form of complementary roles in collective production.

Kasongan aesthetics are formed through collaboration and specialization. Most ceramics are made through a multi-hand process; therefore, a specific product rarely becomes an individual’s work. The initial formation that is often performed by men and the finishing that is commonly performed by women are mutually determined. As a result, the aesthetic that emerges is not a male or female style but a Kasongan aesthetic that is the result of compromises, routines, and unwritten rules operated by the community. Aesthetic negotiations take place through agreements on the minimum thickness, surface fine-roughness standards, symmetrical precision, conformance of shapes to traditional standards, and adjustments based on market needs.

The more dominant affective orientations in female artisans, such as emotional connections through touch and the rhythm of hand gestures, are reflected in subtle and detailed aesthetic choices. Meanwhile, the moral orientation and work ethics of male craftsmen are reflected in a stable and structured form. This shows that aesthetics are expressions of affection: the way materials are perceived is manifested in visual form. Market demand is an important negotiation factor. Customer demand often determines the division of tasks, particularly in motifs and minute details, so that aesthetics are formed not only from the body and emotional experience but also from the rhythm of production, commercial standards, and foreign market preferences.

Overall, the aesthetics of Kasongan ceramics can be understood as a representation of the community identity. Craftsmen realize that they work in a long tradition, so aesthetics is considered something to be maintained as a form of cultural continuity. Kasongan aesthetics are collective, not individual, traditional yet adaptive; contain gender-based differences without creating barriers; and body-based and sensory experience, not just visual design. Aesthetics itself is interpreted as a science that studies the experience of beauty as the highest form of human love for beauty itself ( Magdalena et al., 2022). Thus, the aesthetics of Kasongan ceramics are the result of layered negotiations between bodies, communities, traditions, markets, and emotions.

Table 4 shows that the results of the analysis of observations and interviews show that the ceramic production space in Kasongan not only functions as a workplace but also as a social space where artisans build relationships, form norms, negotiate roles, and produce gender and cultural identities. This is identified through the coding of keywords such as *family*, *habits*, *flexibility*, *no rules*, *cooperation*, and *customer demand*, which are integrated into one major theme: production work as a collectively negotiated social practice. The production process does not take place in a rigid formal structure, but rather in a social space that resembles a family, where the division of tasks, work rhythms, and relationships between individuals is shaped by the habits, experiences, and needs of production. Relationships between craftsmen are carried out through the principles of mutual understanding and politeness, as emphasized by P5 that work patterns are formed from customer habits and requests, not from formal rules. This is in line with the concept of habitus ( Bourdieu, 1990), where practices arise from habits that are embedded through daily social interactions. This concept successfully overcomes the dichotomy of the individual of society, agents of social structure, and determinism of freedom ( Fatmawati & Solikhin, 2020).

**Table 4.** Aesthetics as a result of social negotiation.

Aesthetic aspects	Finding patterns - Male	Finding patterns - Women	Socio-aesthetic implications
Character shapes	Firm, big, sturdy	Smoothness, detail, precision	These two visual orientations merge into the Kasongan style
Working techniques	Intense pressure, efficiency, fast forming	Gradual smoothing, detail correction	Techniques produce differences in texture and style
Motif	Geographical, large structure	Nature motifs, small patterns	Representing complementary roles in design
Role in production	Early formation, structure	Finishing, detailing, coloring	Collective aesthetics are created through collaboration
External factors	Market standard, large order	Detail requests, subtlety	The aesthetics of the form of local–market negotiation

The division of labor in ceramic production is also flexible and not based on strict gender boundaries. Although there is a tendency for certain patterns, for example, women are more in the molding and finishing stages, and men are in heavy work such as *grinding* and detailing, as explained by P1, these patterns are not normative.

“ *Casting Molding) male workers in the grinding and detailing section, female workers in the casting and polishing of ceramic surfaces (finishing*)” (P1, November 17, 2025).

The division of labor depends on skills, physical comfort, and production needs, as emphasized by P4, who rejects the gender-based division of labor and states that work is determined by *skills and job vacancies.*

*“Distribution based on skills and job vacancies”* (P4, November 17, 2025).

Thus, gender is not a structural determinant, but a variable that is constantly negotiated in daily practice. Work solidarity has also emerged as a social capital of production because the gradual nature of ceramic production demands dependence between craftsmen. When there are technical difficulties or delays in order, artisans help each other without a formal leadership structure so that the production space serves as a collective arena that supports the economic sustainability of the community.

Additionally, the Kasongan production room is an arena for gender identity negotiations. In this context, gender identity is produced through bodily practices, social interactions, and production needs, rather than through external rules. P2 and P3’s (2011) statement that all genders can perform at all stages, with physical adjustment as the main consideration, shows fluid gender performativity. Production practices in Kasongan are carried out through two forces that work in parallel: traditions that maintain the continuity of community values and adaptation that allows innovation and response to market changes. P5 explained that work patterns are often determined by customer demand; therefore, the production space becomes a meeting point between local values and external needs. From the perspective of material culture ( Ingold, 2013), such spaces maintain the sustainability of craft because they are not trapped in rigidity but are able to negotiate with change.

Thus, the production space in Kasongan is a dynamic social space that combines kinship relations, flexibility in the division of roles, collective solidarity, gender identity negotiation, and dialectics of tradition and adaptation, all of which form the distinctive character of ceramic production as a social and cultural practice that continues to move.

Table 5 shows that the production space functions as a continuously negotiated social space, where work relationships are built like a family through warmth and non-formal habits thus giving birth to strong social capital and a sense of belonging. The division of labor is flexible based on skills and needs rather than gender, asserting that gender identity is fluid and emerges through everyday practice. Solidarity is the foundation of production sustainability because workers help each other in dealing with workloads and personal problems. In addition, the creative process takes place through negotiations between tradition and adaptation to market demand, showing that aesthetics and production are dynamic and responsive to social and economic contexts.

**Table 5.** Production space as a negotiated social space.

Subtheme	Pattern of findings	Social implications
Family workspace	Warm relationships, full of habits, without formal rules	High social capital, sense of belonging
Flexible division of work	Determined by skills and needs, not gender	Gender is negotiable and situational
Work solidarity	Help each other in production and face difficulties	The sustainability of production depends on social capital
Gender identity negotiation	Gender shows up through practice, not rules	Fluid and adaptive gender identity
Tradition–adaptation	Tradition is maintained but adapts to the order	Aesthetics and production are dynamic

## Discussion

The results of this study show that the practice of Kasongan ceramic production is a cultural space that allows the body, materials, aesthetics, and social relations to work simultaneously and form each other. Ceramic production is not only understood as a technical process but also as a living and continuously negotiated ecosystem of knowledge. The following discussion integrates the findings of this field with key theories and presents analytical arguments that clarify the position of this research in craft discourse, material culture, and gender studies.

Field findings confirm that a craftsman’s body functions as the main medium of knowledge production. All informants refer to body parts, physical intensity, rhythm, and endurance as central factors in their work, which determine the quality of production (P3, P4, Hopefully, November 17, 2025). The emphasis on the body in the production process proves that the skill of making ceramics is an embodied form of knowledge, as explained by Sennett (2008), which is knowledge embedded through repetitive actions and direct experience, and not solely understood cognitively or theoretically. This kind of knowledge grows from the body’s constant engagement with materials, tools, and work rhythms, so that skills cannot be transferred instantly through verbal instruction, but must be experienced and trained through practice.

The contrast between the body strategies of male and female craftspeople demonstrates how skills are built around the necessity of daily labor. Macro manœuvres, pressure strengths, and physical abilities are natural from the heavier tasks of the work-intensive stages of production. Meanwhile, women’s sex skills grow with step-by-step tiny movements, slow rhythms, or details of facial appearance in places such as casting, finishing, and looking for a perfect face. It is a hierarchy of neither expertise nor dominance of one group over another, but two lines in the flesh that complement each other and guarantee the continuity of the general process. When it comes to the division of labor, it is daily nugiating to reach a point where the workers on any craft know both their limits and possibilities in relation to their bodies, who also have certain physical conditions and skills.

The practice of Kasongan ceramics shows that body knowledge is not only a part of production techniques, but also the foundation of the sustainability of traditions. The body becomes a space in which the memory of techniques, work values, and social habits is stored, reproduced, and inherited. Technical knowledge such as finger pressure levels, turntable rotation speed, or sensitivity to changes in clay moisture cannot be fully recorded in a document but live in the bodies of craftsmen who continue to use it every day. Thus, the tradition of craft survives not because of written documentation or formal curriculum but because of the continuity of embodied knowledge inherited through direct learning between generations through seeing, imitating, feeling, and caring for the relationship between body, material, and community. Artisans symbolically and emotionally understand clay as a living entity. Expressions such as “the land is alive” and “the land is alive” (P6; P4, November 17, 2025) shows that the relationship between craftsmen and materials is not a utilitarian relationship or just an instrument of production, but an affective and existential relationship. Materials are treated not as inanimate objects but as partners that have their own power, rhythm, and character. This is consistent with Ingold’s (2013) argument in material engagement theory that materials are active and never passive; they shape human reflections and perceptions of sensibility in work activities.

In everyday life, material relations in terms of affective relations seem to have gender experience-related differences. Women artisans often read the land as an affective site where they quieten down and sort their feelings in response to the demands of domestic and social life. Instead, male workers tended to understand land in the moral and spiritual dimensions of work culture, responsibility, and discipline. This difference in the mode of material interpretation reveals that the love of land is part of gender performativity developed through embodied practices, according to Butler (2011). This affective quality, the relationship between craftsmen and their ‘charming material,’ continues to be a significant element in explaining the longevity of Kasongan ceramic practices. When material is treated as an emotional, moral, and spiritual value, the production process is no longer simply understood as income-generating work but as an integral part of identity, social life, and cultural sustainability. Thus, the sustainability of the Kasongan ceramic tradition is not only supported by economic and market factors but also by the affective dimension that binds the craftsman to the material and to his community.

The aesthetics of Kasongan ceramics do not emerge as individual expressions but rather as a result of complex social negotiations between body strategies, gender relations, and external demands. Differences in aesthetic tendencies between male and female artisans, such as firm structure and large volume in male work and subtle details and small motifs in female work, are related to different embodied experiences (P2, Hopefully, P5, November 17, 2025). Aesthetic choice reflects the relationship between the body and material, and the gender-based division of work that has been formed and inherited in the tradition of Kasongan production.

In everyday production practice, ceramic work is not borne by the hands of one manufacturer. This process is a complementary collaboration. Male craftsmen shape the basic structure and main construction, while women refine details, give texture, and add visual accuracy that embellishes the final look. Through such collaboration, the characteristics of Kasongan ceramics can be maintained consistently, not because of the dominance of the identity of a single artist but because of collective standards that are inherited across generations and are always negotiated together. Market demand also plays a vital role in shaping the aesthetics of Kasongan. Craftsmen must adjust the motifs, sizes, precision, and production quantities according to the needs of buyers as well as the development of global design trends. Consumer product matching in the market In the market, consumers request commodities according to their specifications or requirements and also valuate the goods that are offered. The aesthetic shifts as demand shifts, but remains tied to the base of local traditions. Terzo, un tale procedere mostra come l’estetica di Kasongan sia la scontrosa crocevia tra i valori locali e le pressioni del mercato internazionale.

**Figure 5.**
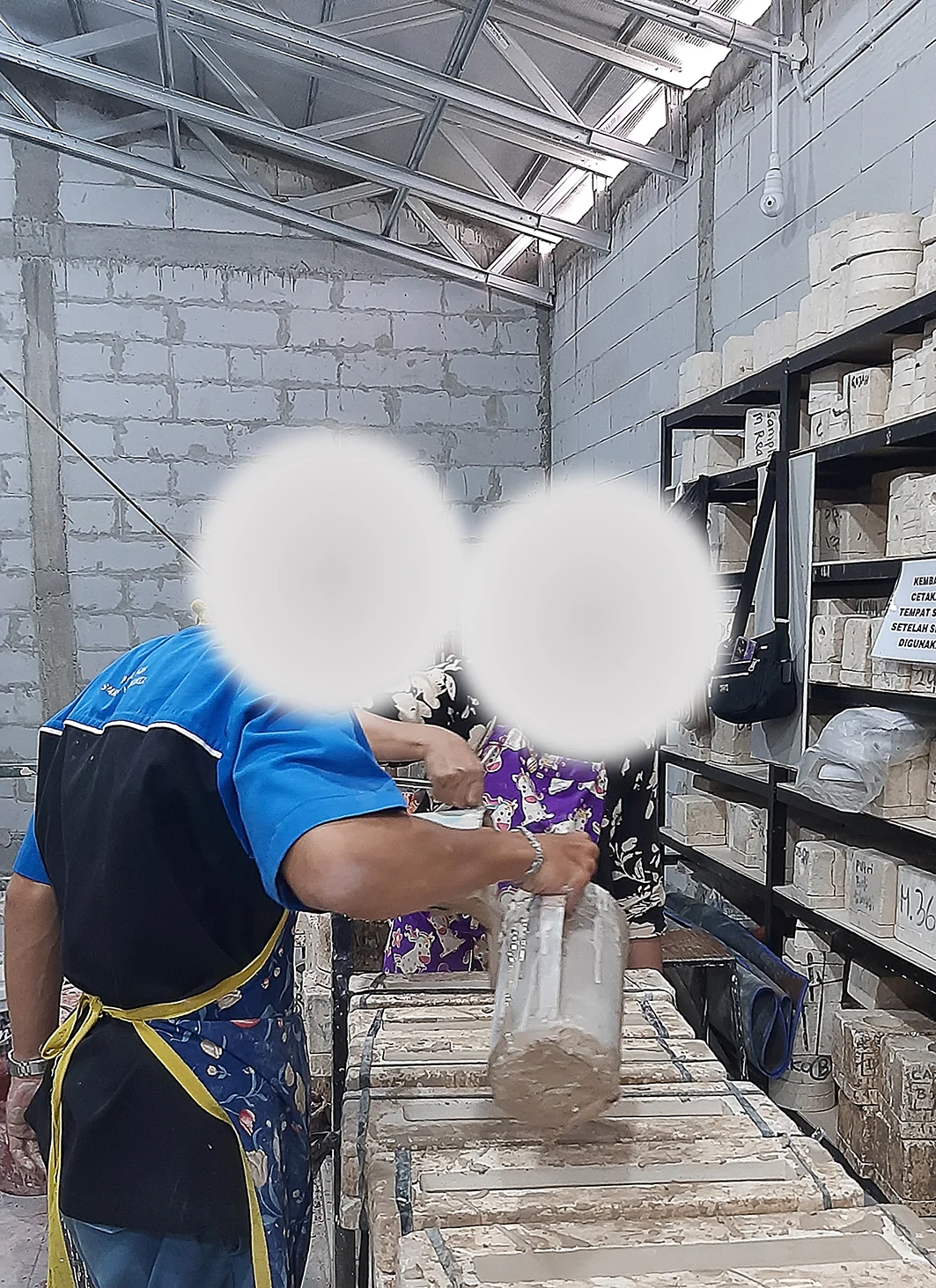
Ceramic Molding Process.

**Figure 6.**
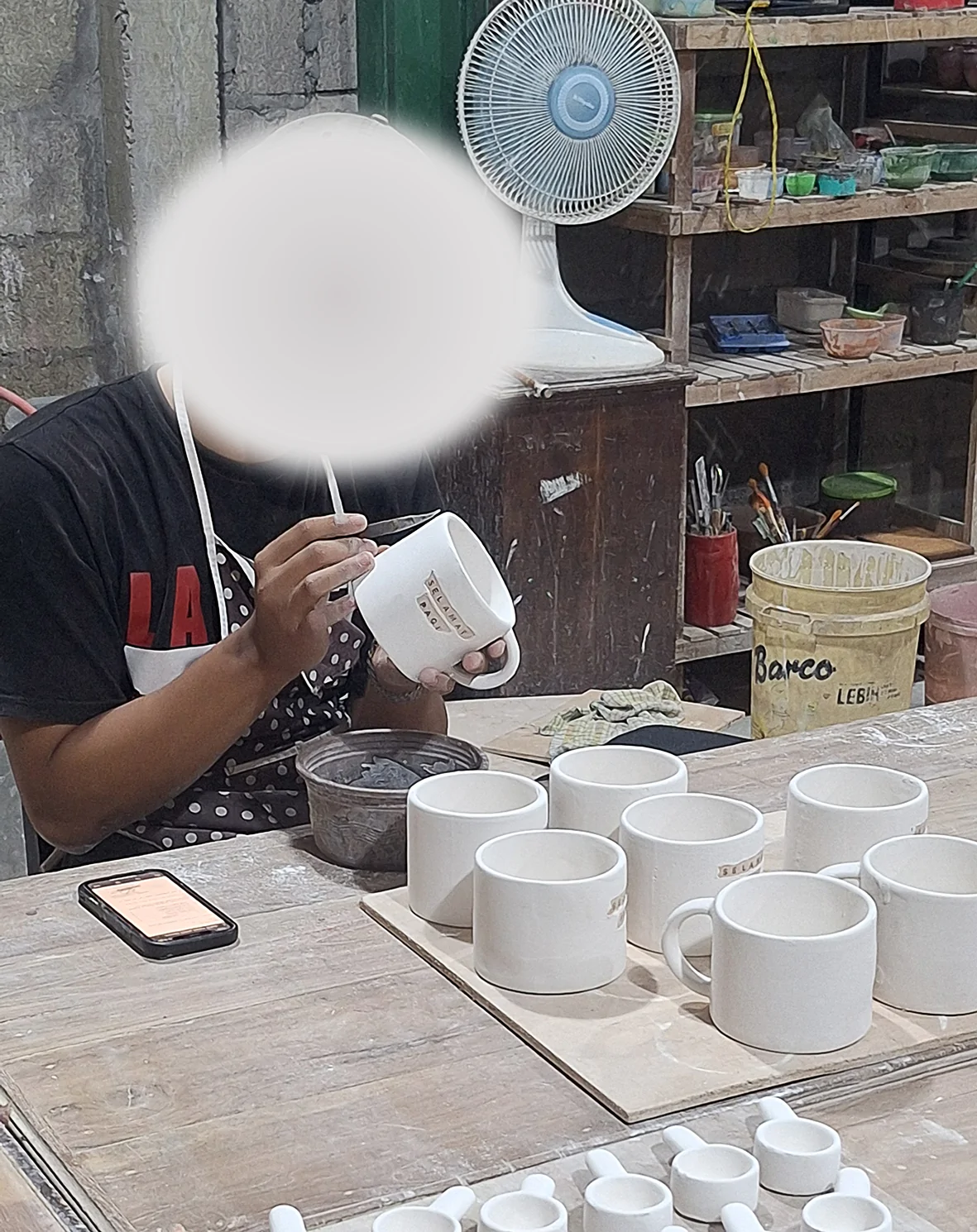
Manual surface refinement of ceramic vessels prior to firing.

The results indicated that the production space of Kasongan ceramics acts as a social presence, much like a family. Relationships among the aseres are not dictated by formal hierarchies but by patterns of working together, solidarity, and production needs ( Figure 5 and Figure 6). There are no strict labor divisions.” Some key informants also described that the work was organized on the basis of capability and experience, but not based on gender categories (P4; P2). P3, November 17, 2025. This open and flexible way of working indicates that the social structure of ceramic production is built through daily practice and interaction.

This situation also reinforces gender flexibility in Kasongan. The roles of men and women can be interchangeable, depending on the production conditions and requirements. Women can perform classical constructions if needed; men can go into finishing or whatever other classic woman’s work there is. In Butler’s (2011) analysis, the Kasongan production space can be regarded as a continuous gender performing space where identity performativity also takes place, in which such an identity is reproduced through repetition rather than following rules from the outside.

One of the most important things is solidarity, which facilitates the whole thing to work seamlessly because we rely so much on each other between all stages. The interconnectedness of processes transforms the production space into more than just a work area, but also a social and cultural environment, where values, ethics, and collective responsibilities converge. For this reason, collective labor forms the basis of production and preserves the unity of artisans. Therefore, the Kasongan production space shows an egalitarian social model that develops from real work needs and not from formal gender equality ideologies. This condition shows that the transformation of the gender structure can be derived from natural and gradual daily practices, not through policy interventions. Gender flexibility in Kasongan is a form of cultural transformation that occurs organically through shared experiences and continuity of tradition.

The discussion above shows that Kasongan ceramic production operates as a complex and interrelated knowledge ecosystem. Such an ecosystem is composed of body, material, collective, and gender knowledge, all of which work simultaneously in shaping the sustainability of traditions. Such knowledge does not arise through formal processes, such as written curriculum or certification systems, but rather through embodied practices rooted in sensory experiences and physical interactions between the craftsman’s body and the clay material. In the framework of the theory of material engagement proposed by Tim Ingold (2013), the relationship between humans and material is not an instrumental one-way relationship but an interactive relationship that shapes the way we think, feel, and act. Materials are understood as agents that participate in the process of knowledge production; therefore, the production of Kasongan ceramics can be seen as a co-evolutionary process between the body and the material. Knowledge of work rhythm, hand pressure, soil moisture, and physical endurance cannot be transmitted through verbal instruction but rather through direct experience and continuous bodily engagement. In this context, the body is an archive of knowledge and embodied experience becomes the main mechanism of the production and inheritance of traditions.

**Figure 7.**
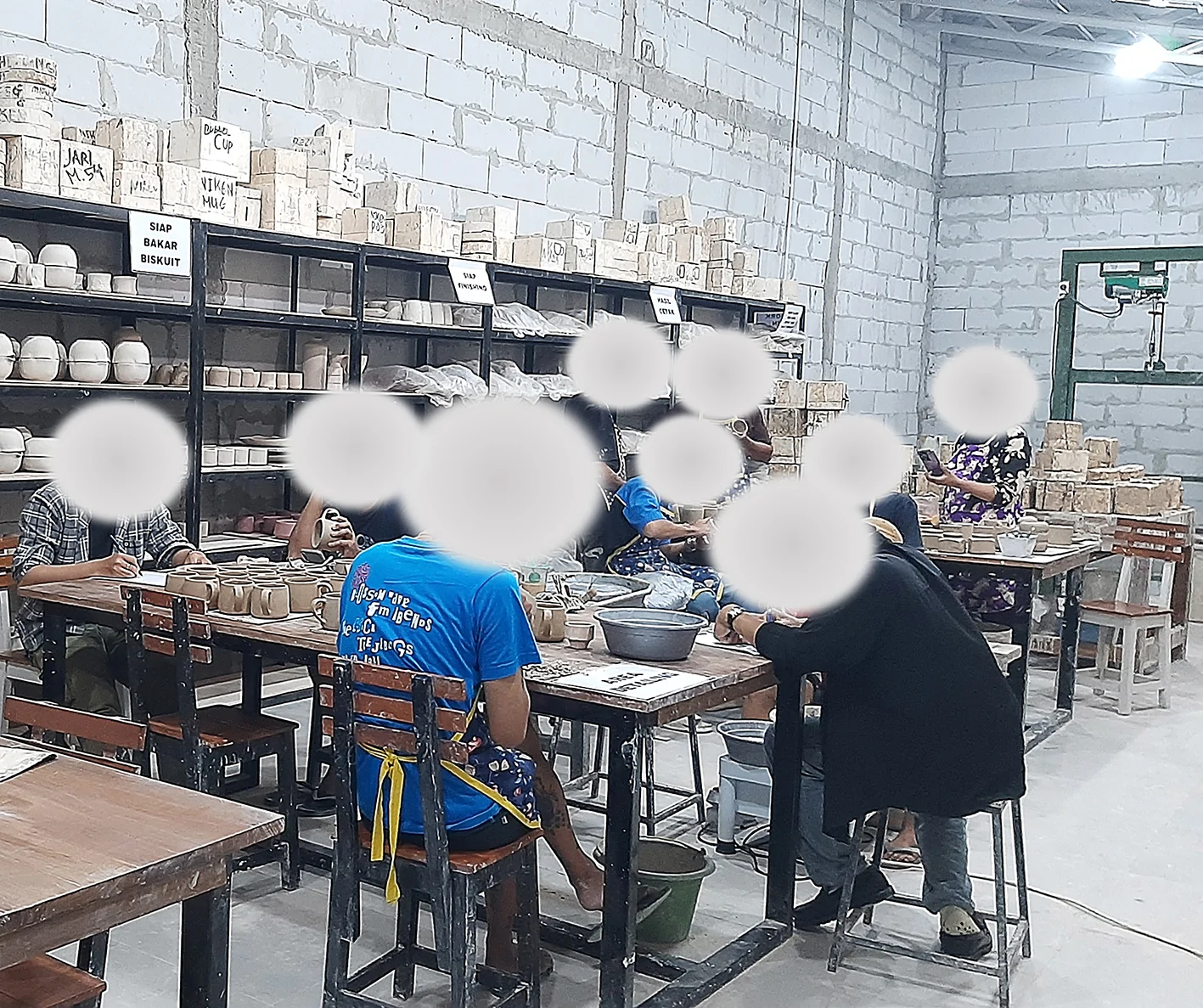
Workplaces that are occupied by both male and female genders.

Collective knowledge in Kasongan production is inseparable from the surrounding social structure. Tradition is maintained not through formal structures, but through social negotiation and participatory learning. Polanyi (2009) emphasized that tacit knowledge cannot be fully articulated in words but rather lived through action. This explains why Kasongan artisans state that they “learn from seeing and doing”, not from the curriculum. The learning process occurs through repetition, observation, adjustment, and correction, which are continuously occurring in the production space. Gender knowledge in Kasongan production is not static. The apparent division of roles between male and female craftsmen is not the result of rigid patriarchal structures, but the historical consequences of the formation of different bodies through work habits. Butler (2011) explains that gender is produced through performativity, which is a repetitive action and produces a natural-looking form of identity. Thus, gender flexibility in Kasongan shows how gender identity continues to be negotiated through work practice. When women take on structural roles or men work on minute details, they disrupt normative gender boundaries and show that gender is not a fixed biological category but rather an open and changing social practice ( Figure 7).

Moreover, the connection between the body, materials, and community makes the Kasongan production space a social arena where cultural values, work ethics, and gender identity are negotiated every day. Solidarity and cooperation are key to successful production, especially because each phase of the operation depends on the others. This is consistent with Lave’s (1991) theory of communities of practice, which argues that learning and knowledge do not come from formal educational organizations but are generated by participating in community practice. The Kasongan workshop space is a community of practice, where tradition is not replicated through doctrine, but rather through collective participation. This kind of understanding shows that the sustainability of tradition in Kasongan cannot be understood only from the side of administrative cultural resistance, but also from flexible culture that enables continuous negotiation between past and present. Kasongan persists because it can constantly renegotiate aesthetic identity and mode of production in relation to shifting global markets, consumer demand dynamics, and the internal evolution of the artisan community. Traditions are retained by adaptation, not stasis.

In this context, Kasongan ceramics constitute a live system of culture and knowledge that is still evolving and transforming in encounters with the body, materials, markets, and communities. The durability of Kasongan is not because the traditional form is permanent but because of the knowledge that can be circulated again and reinterpreted according to a continuously changing context. Thus, the making of Kasongan ceramics is both exemplary and symptomatic of the struggle between the past and present, tradition and modernity, practice in the body, and capital economy that plagues delights notifies some of the material cultures attempts to conspire toward social negotiations over identity.

## Conclusion

This study reveals that the experience of Kasongan ceramic production is a cognitive ecosystem composed of body, materials, and social ties. A craftsman’s body becomes the principal medium for forming skills through experience, rhythm memory, and continuous contact with clay. Physical differences in body strategy between male and female craftsmen are also a specialization of work that is complementary to the structure of traditional ceramic production. The affective relationship between craftsmen and clay shows that materials in ceramic practices are not understood as purely technical objects. Clay is treated as a living, meaningful, emotional, spiritual entity. This relationship influences the way artisans work, feel, and produce aesthetics. Thus, materials play an active role in the creative process and contribute to the sustainability of Kasongan craft tradition.

Ultimately, the aesthetics of Kasongan ceramics have been proven to be the result of social negotiations between the body, gender, skills, community traditions, and market demands. Works are not born as individual expressions but as a result of the collaboration of various stages of production involving many hands. The difference in the aesthetic orientation of men and women produces a typical visual characteristic of Kasongan that is stable but still adaptive to consumer needs, so that aesthetics becomes a mediating space between local identity and economic dynamics. The Kasongan ceramic production space functions as a flexible and non-hierarchical social space resembling a family structure. The division of labor is adaptive, ability-based, and not limited by gender. Solidarity, habits, and collective values are the foundation of community sustainability. All these findings confirm that the sustainability of Kasongan craft arts rests on the ability of its community to continuously negotiate traditions and adaptations, making ceramics a form of material culture that is alive and constantly evolving.

## Ethics and consent

This study did not require formal ethical approval, as it constitutes a non-invasive qualitative study within the field of arts and humanities, and the author’s affiliated institution does not mandate ethical review by an Institutional Review Board for this type of research. Nevertheless, ethical principles were rigorously upheld throughout the entire research process. In addition, an institutional statement from the head of the author’s department is attached as supporting documentation, confirming that the study has been conducted in accordance with applicable institutional regulations.

All participants took part in the study voluntarily and provided informed consent prior to their participation. Participants were fully informed about the objectives of the study, the use of interview data, and their right to withdraw from the research at any stage without any consequences. Participant anonymity and data confidentiality were strictly maintained. Informed consent was obtained from all participants prior to data collection. All participants were adults (≥18 years old). Participants were provided with detailed information regarding the objectives of the study, the voluntary nature of participation, and assurances of data confidentiality. Verbal consent was obtained to ensure that all participants could understand the research information equally, without being hindered by reading ability or comprehension of written documents.

## Data Availability

The data supporting the findings of this study are not publicly available due to ethical and privacy considerations, as they contain detailed qualitative information derived from in depth interviews that may indirectly identify participants. The study protocol and data sharing procedure were reviewed and approved by the institutional ethics committee, which restricts public deposition of the dataset to protect participant confidentiality in accordance with the consent agreement. Researchers who meet the criteria for access to confidential data may request the dataset from the corresponding author via email at nadiasigi@mail.unnes.ac.id. Access will be granted for academic purposes only, subject to ethical approval, a formal request describing the intended use of the data, and an agreement to maintain participant confidentiality and not attempt re identification. Figshare. Extended data. https://doi.org/10.6084/m9.figshare.31343179 ( Prameswari, 2026). This project contains the following underlying data:
• Extended Data File 1. (The primary dataset containing the research data used in the study analysis). Data is available under the terms of the Creative Commons Attribution 4.0 International (CC BY 4.0) license.
