# Supplementary material for: Clay as Space for Negotiation: Gender Relations in Traditional Ceramic Production
Source: F1000Res. 2026 May 19;15:756. [Version 1] doi: 10.12688/f1000research.177142.1 (PMC13560520; doi:10.12688/f1000research.177142.1)
Supplement: Peer review history

# Reviewer report: Reviewer response for version 1

**DOI:** 10.5256/f1000research.195312.r501917

**Published:** 2026-08-21

**Authors:** Roziani Mat Nashir

**Affiliations:** 1 Faculty of Art and Design, Universiti Teknologi MARA - Cawangan Kelantan, Machang, Kelantan, Malaysia

The manuscript presents an interesting and meaningful investigation of gender relations, embodied knowledge, and material engagement within traditional ceramic production in Kasongan, Yogyakarta. The study is well positioned within the fields of craft studies, material culture, and gender studies, and its focus on ceramic production as a space of social negotiation provides a valuable perspective. The qualitative research design is appropriate, and the combination of interviews, participatory observation, and visual documentation provides a suitable basis for interpreting artisans’ experiences and everyday production practices. The findings clearly demonstrate how bodily knowledge, relationships with clay, flexible gender roles, collective work, and market demands interact in shaping ceramic production and its aesthetics. The discussion also connects the empirical findings effectively with the theoretical framework and emphasises the cultural value and sustainability of Kasongan ceramic traditions. Overall, the manuscript makes a relevant and original contribution to the field. I consider the article suitable for indexation and recommend approval in its present form.

Is the work clearly and accurately presented and does it cite the current literature?

Yes

If applicable, is the statistical analysis and its interpretation appropriate?

Not applicable

Are all the source data underlying the results available to ensure full reproducibility?

Yes

Is the study design appropriate and is the work technically sound?

Yes

Are the conclusions drawn adequately supported by the results?

Yes

Are sufficient details of methods and analysis provided to allow replication by others?

Yes

Reviewer Expertise:

visual communication, graphic design, visual branding

I confirm that I have read this submission and believe that I have an appropriate level of expertise to confirm that it is of an acceptable scientific standard.

# Reviewer report: Reviewer response for version 1

**DOI:** 10.5256/f1000research.195312.r496516

**Published:** 2026-07-03

**Authors:** Anke Hein

**Affiliations:** 1 University of Oxford, Oxford, England, UK

This is a very interesting article investigating the role of gender in ceramic production practices in one specific potting community, namely Kasongan, Yogyakarta, based on ethnographic research. The methods used include semi-structured interviews, participatory observations, and visual documentation of six potters. The study is embedded suitably in conceptual frameworks on the connections between knowledge, body, and identity. The approach and analyses are clear, the observations are well-described and presented in a transparent way, and the inferences are well-supported. All in all, the research is well-executed and well-presented and ready for indexing.

Is the work clearly and accurately presented and does it cite the current literature?

Yes

If applicable, is the statistical analysis and its interpretation appropriate?

Not applicable

Are all the source data underlying the results available to ensure full reproducibility?

Yes

Is the study design appropriate and is the work technically sound?

Yes

Are the conclusions drawn adequately supported by the results?

Yes

Are sufficient details of methods and analysis provided to allow replication by others?

Yes

Reviewer Expertise:

archaeology, ceramic analysis, ethnoarchaeology, Chinese archaeology and history

I confirm that I have read this submission and believe that I have an appropriate level of expertise to confirm that it is of an acceptable scientific standard.
